# Gestational Obstructive Sleep Apnea: From Pathophysiological Mechanisms to Maternal-Fetal Outcomes

**DOI:** 10.7759/cureus.105659

**Published:** 2026-03-22

**Authors:** Mohammed Al-Hamas, Syedakhudajja Zoha, Siti Abdulrahman, Alexander Gate, Awaisilyas Mohammed, Ahmad Diab, Christos Charalambous, Ali Abbas, Abdel Motaleb Moukalled, Megha Vijay Kumar

**Affiliations:** 1 Acute Internal Medicine, Hull York Medical School, Hull, GBR; 2 Internal Medicine, United Lincolnshire Teaching Hospitals NHS Trust, Lincoln, GBR; 3 General Practice, GP Trainee (ST2), Nottingham GP Specialist Training Programme, Nottingham, GBR

**Keywords:** high-risk pregnancy, non- invasive ventilation, pregnancy induced hypertension (pih), pregnancy-related complications, risk factors for obstructive sleep apnea (osa)

## Abstract

Obstructive sleep apnea (OSA) during pregnancy is increasingly recognized as a clinically significant condition associated with maternal cardiovascular and metabolic complications, as well as adverse fetal outcomes. Physiological changes in pregnancy, including weight gain, airway edema, reduced functional residual capacity, and altered ventilatory control, can exacerbate or unmask sleep-disordered breathing. Emerging evidence suggests associations between gestational OSA and hypertensive disorders of pregnancy, gestational diabetes mellitus, cesarean delivery, and preterm birth.

Continuous positive airway pressure (CPAP) remains the primary therapy for moderate to severe disease, while noninvasive ventilation may be required in selected ventilatory failure phenotypes, such as obesity hypoventilation syndrome. Mandibular advancement devices (MADs) may offer an alternative in carefully selected patients with mild disease or CPAP intolerance. This review synthesizes current evidence regarding the epidemiology, pathophysiology, maternal-fetal implications, diagnostic strategies, and evolving treatment approaches for OSA in pregnancy. Particular emphasis is placed on risk-stratified screening, phenotype-guided therapy, and the importance of multidisciplinary management throughout the antenatal and postpartum periods.

## Introduction and background

Obstructive sleep apnea (OSA) is characterized by recurrent upper-airway obstruction during sleep, resulting in intermittent hypoxemia, intrathoracic pressure swings, sympathetic activation, and sleep fragmentation [1,2]. In non-pregnant populations, OSA is associated with hypertension, impaired glucose metabolism, endothelial dysfunction, and increased cardiovascular risk, reflecting a pathophysiological cascade driven by repeated hypoxia-reoxygenation and neurohumoral activation [2]. These mechanisms are particularly relevant in pregnancy, where physiological adaptation can unmask pre-existing sleep-disordered breathing or worsen mild disease, potentially amplifying cardiometabolic vulnerability.

Pregnancy promotes OSA through several converging mechanisms. Progressive gestational weight gain, rostral fluid shifts, and mucosal edema increase upper-airway collapsibility, while diaphragmatic elevation and reduced functional residual capacity decrease oxygen reserve and increase the severity of desaturation during obstructive events [3]. At the same time, symptoms such as fatigue, insomnia, and fragmented sleep are common in otherwise uncomplicated pregnancy, which reduces the specificity of symptom-based screening and can delay diagnosis [4]. Importantly, disturbed sleep in pregnancy, independent of OSA, has been linked to inflammatory activation and adverse perinatal signals, underscoring the need to distinguish general sleep disruption from OSA-specific obstructive physiology [5].

Clinically, the growing importance of gestational OSA relates to its association with major obstetric complications. Systematic reviews and cohort studies have reported increased risks of hypertensive disorders of pregnancy and gestational diabetes mellitus among individuals with sleep-disordered breathing, although residual confounding from obesity and baseline comorbidity remains a limitation of the available evidence [6,7]. While continuous positive airway pressure (CPAP) remains the principal therapy for clinically significant disease, and emerging evidence suggests possible benefit for hypertensive outcomes, high-quality pregnancy-focused trials remain limited [8]. This review synthesizes contemporary evidence on epidemiology, mechanistic pathways, maternal-fetal outcomes, and diagnostic and management strategies for OSA in pregnancy, with emphasis on risk-stratified assessment, phenotype-guided treatment selection, and multidisciplinary care across the antenatal, peripartum, and postpartum periods.

## Review

Epidemiology and natural history

The prevalence of OSA in pregnancy varies by population risk profile, gestational age at assessment, and the diagnostic method used. Meta-analytic evidence suggests that objectively assessed sleep-disordered breathing affects a meaningful proportion of pregnant patients, with prevalence rising in cohorts enriched for obesity and cardiometabolic risk factors [1]. Notably, administrative coding and symptom-only ascertainment likely underestimate the true disease burden, as many pregnant individuals experience nonspecific sleep symptoms that are not reliably discriminatory for OSA [4].

The clinical course of OSA across pregnancy is dynamic rather than static. Disease may predate pregnancy and become more apparent as gestation progresses, or it may develop de novo, particularly in the third trimester, when upper-airway collapsibility and vulnerability to oxygen desaturation increase [3]. Consequently, the absence of symptoms in early pregnancy does not exclude clinically relevant OSA later in gestation, especially when weight gain, edema, or metabolic complications accumulate [3,6,7].

Risk factors associated with gestational OSA overlap substantially with those for adverse obstetric outcomes. Obesity, chronic hypertension, insulin resistance, and prior hypertensive disorders of pregnancy increase the probability of OSA and may also amplify downstream risk once sleep-disordered breathing is present [1,6,7]. In practical terms, gestational OSA often functions as both a potential contributor to risk and a marker of a broader cardiometabolic phenotype, which should guide how clinicians interpret associations and choose interventions [6,7].

Pathophysiology and mechanistic links to obstetric morbidity

OSA produces systemic physiological stress through repeated cycles of airway obstruction, hypoxemia and reoxygenation, intrathoracic pressure swings, and arousal. These events promote sympathetic activation, oxidative stress, endothelial dysfunction, and inflammatory signaling, mechanisms strongly linked to cardiometabolic morbidity in non-pregnant populations [2]. These processes provide biological plausibility for associations between OSA and hypertensive or metabolic complications in pregnancy [6,7].

Pregnancy-specific physiology may intensify these mechanistic pathways. Reduced functional residual capacity diminishes oxygen reserve, so even short obstructive events can result in deeper or more frequent desaturation [3]. Upper-airway mucosal edema and fluid shifts increase collapsibility, while increased oxygen consumption and altered ventilatory control may amplify physiological stress during sleep [3]. Together, these changes help explain why pregnancy can unmask mild OSA or worsen pre-existing disease, particularly in the context of obesity and cardiometabolic vulnerability [1,3].

It is also critical to distinguish OSA from general sleep disturbance. Insomnia, short sleep duration, and fragmented sleep are common in pregnancy and are independently associated with inflammatory activation and adverse perinatal signals [5]. However, these sleep problems are not synonymous with OSA, which is defined by obstructive respiratory events and intermittent hypoxemia. This distinction matters because the mechanisms, diagnostic tests, and treatment options differ [2,5].

Maternal outcomes

Hypertensive Disorders of Pregnancy

Across systematic reviews and observational cohorts, maternal sleep-disordered breathing has been consistently associated with hypertensive disorders of pregnancy, including gestational hypertension and preeclampsia [6,7]. The association is biologically credible: intermittent hypoxemia and sympathetic activation can impair vascular function and increase vasoconstrictor tone, while oxidative stress and endothelial dysfunction may contribute to the vascular phenotype that characterizes hypertensive pregnancy disorders [2,6,7].

Nevertheless, interpretation should remain appropriately cautious. Obesity and baseline cardiometabolic disease are major confounders and may partially mediate the relationship between OSA and hypertensive outcomes. The most defensible clinical framing is that OSA identifies a higher-risk phenotype and may plausibly contribute to hypertensive pathophysiology, even when absolute causality is difficult to isolate in observational designs [6,7].

Gestational Diabetes Mellitus and Metabolic Outcomes

OSA has also been associated with gestational diabetes mellitus (GDM) and impaired glycemic control in pregnancy [6,7]. Potential mechanisms include sympathetic activation, oxidative stress, and sleep fragmentation, factors that can promote insulin resistance and impair glucose regulation [2]. These pathways align with broader evidence linking OSA with metabolic disease outside pregnancy, supporting biological plausibility [2].

As with hypertension, confounding is important. Obesity is a shared risk factor for both OSA and GDM, and the degree to which OSA independently worsens glycemic outcomes remains incompletely defined. Clinically, however, the coexistence of GDM and suspected OSA should heighten suspicion for sleep-disordered breathing and prompt objective evaluation, particularly when symptoms or nocturnal hypoxemia are prominent [6,7].

Delivery Outcomes and Broader Maternal Morbidity

Some studies report associations between gestational sleep-disordered breathing and increased likelihood of cesarean delivery, operative intervention, and preterm birth, although effect sizes vary and consistency is weaker than for hypertensive and metabolic outcomes [1,6]. This variability likely reflects heterogeneous populations, differing disease definitions, and residual confounding.

A clinically pragmatic interpretation is that gestational OSA is part of a broader risk cluster that can influence labor and delivery planning (e.g., anesthesia considerations, postoperative monitoring) rather than a single direct cause of operative delivery. The presence of clinically significant OSA should therefore prompt anticipatory planning without overstating certainty about specific delivery endpoints [3,6].

Fetal and neonatal outcomes

Evidence regarding fetal and neonatal outcomes is more heterogeneous than for maternal cardiometabolic outcomes. Some studies suggest associations with preterm birth, altered fetal growth trajectories, or neonatal complications, while others show attenuation after adjustment for maternal obesity and comorbidity [1,6]. Differences in outcome definitions, timing of OSA assessment, and severity distributions likely contribute to inconsistent findings.

Despite uncertainty, maternal physiology provides plausible pathways for fetal impact. Maternal intermittent hypoxemia, vascular dysfunction, and metabolic dysregulation may influence placental perfusion and the fetal growth environment, particularly in higher-severity disease or in patients with coexisting hypertensive or metabolic complications [2,6]. Therefore, while certainty regarding neonatal endpoints is limited, clinicians should still treat gestational OSA as clinically meaningful, especially in high-risk phenotypes [1,6].

Continuous positive airway pressure

CPAP remains the cornerstone therapy for moderate to severe OSA and for symptomatic patients with clinically significant disease. CPAP mechanically splints the upper airway, reducing obstructive events and improving nocturnal oxygenation, thereby addressing the central pathophysiology of OSA [2].

Recent meta-analytic evidence suggests that CPAP treatment in pregnancy may reduce hypertensive adverse outcomes, although certainty remains limited by heterogeneity, small-study effects, and variable adherence [8]. Earlier physiological work in preeclampsia demonstrated that nocturnal CPAP can mitigate hemodynamic disturbances, strengthening biological plausibility for improvements in blood pressure-related endpoints [9]. Pilot randomized trial frameworks have explored CPAP’s potential influence on glycemic control in gestational diabetes, supporting the rationale for larger outcome-focused trials [10].

In practice, CPAP should be framed as the best-supported therapy for clinically significant OSA in pregnancy, with benefit most defensibly anchored in improvement of obstructive events and oxygenation. Claims regarding universal improvement across all obstetric endpoints should remain proportional to the quality and maturity of the evidence base [8-10].

Screening, diagnosis, and phenotyping

Accurate diagnosis is challenging because common pregnancy symptoms overlap with OSA presentations. Screening questionnaires have variable performance in pregnant populations and should not be used as stand-alone rule-out tools in individuals with high-risk phenotypes [11]. Therefore, a targeted case-finding approach is often more clinically efficient than universal screening, prioritizing objective testing in those with obesity, chronic hypertension, GDM, witnessed apnea, habitual loud snoring, or disproportionate daytime sleepiness [1,6,7,11].

Polysomnography remains the diagnostic reference standard, particularly when hypoventilation, complex sleep pathology, or alternative diagnoses are suspected [3]. Home sleep apnea testing may facilitate diagnosis where access to polysomnography is limited, but results should be interpreted cautiously, particularly if clinical suspicion remains high despite a borderline result [3,11]. In pregnancy, physiological changes may also affect the relationship between apnea-hypopnea index and hypoxemia burden, making clinical context essential.

Phenotyping is important because treatment selection may depend not only on the number of obstructive events but also on oxygenation burden, comorbid conditions, and the presence of ventilatory failure physiology. This is particularly relevant when persistent hypoxemia, daytime hypercapnia, or severe obesity suggests physiology beyond uncomplicated OSA [3,11]. A phenotype-informed approach supports escalation pathways (e.g., CPAP vs. bilevel support) and helps identify patients who may require closer antenatal and peripartum monitoring.

Non-invasive ventilation and ventilatory failure phenotypes

A subset of pregnant patients exhibits ventilatory failure phenotypes such as obesity hypoventilation syndrome (OHS) or mixed sleep-related hypoventilation. In these cases, CPAP alone may be insufficient, and bilevel positive airway pressure or other non-invasive ventilation strategies may be required to address hypercapnia and persistent nocturnal hypoxemia [12]. Pregnancy-related reductions in oxygen reserve can further magnify clinical vulnerability in these patients, particularly during sedation, labor, or postoperative periods.

Pregnancy-specific evidence remains limited, but indexed case-based literature highlights practical challenges and management principles for OHS during labor and delivery, including diagnosis, ventilatory support optimization, and coordinated peripartum planning [12]. Clinically, these patients warrant early respiratory/sleep specialist involvement, structured escalation criteria, and enhanced monitoring strategies. The central practical point is that “gestational OSA” is not a uniform entity, and identification of ventilatory failure physiology should change management intensity and modality [12].

Mandibular advancement devices

Mandibular advancement devices (MADs) offer a non-mask alternative for selected patients with mild to moderate OSA or those intolerant of CPAP. Pregnancy-specific pilot evidence supports feasibility, objective adherence measurement, and improvement in sleep-disordered breathing indices in selected pregnant patients using mandibular advancement splints [13]. For patients who decline CPAP, or who cannot tolerate CPAP despite troubleshooting, MADs may provide a pragmatic option within a monitored care pathway.

However, the pregnancy-specific evidence base remains limited by small sample sizes and a lack of robust obstetric endpoint data. MADs should therefore be framed as individualized alternatives for selected phenotypes, rather than replacements for CPAP in moderate to severe disease, particularly in those with significant nocturnal hypoxemia or cardiometabolic complications [13]. Follow-up should include symptom reassessment and, where available, repeat objective testing to confirm adequate treatment response.

Peripartum and postpartum considerations

OSA carries peripartum implications due to increased risk of difficult airway management, opioid sensitivity, and perioperative hypoxemia, especially in severe OSA or ventilatory failure phenotypes [3,6,12]. Anticipatory planning includes documenting OSA status in the obstetric record, early anesthesia input, access to the patient’s positive airway pressure device during hospital admission when appropriate, and escalation of monitoring for higher-risk phenotypes [3,6]. Where ventilatory failure is suspected or confirmed, management should prioritize respiratory specialist involvement and careful avoidance of respiratory depressant exposures when feasible [12].

Postpartum reassessment is important because OSA severity may improve after delivery as pregnancy-related airway and respiratory changes resolve, but persistent disease is common in patients with obesity or pre-existing cardiometabolic risk. A structured postpartum pathway should include symptom review, repeat objective testing when indicated, and longer-term cardiometabolic risk mitigation, particularly for individuals with hypertensive pregnancy disorders or GDM, where ongoing risk is elevated beyond pregnancy itself [1,6,7].

## Conclusions

OSA in pregnancy is underrecognized and is consistently associated with hypertensive disorders of pregnancy and GDM in contemporary observational evidence. A phenotype-informed approach is likely to provide the greatest clinical value: CPAP should remain first-line therapy for moderate to severe disease, non-invasive ventilation should be considered for ventilatory failure phenotypes such as obesity hypoventilation syndrome, and mandibular advancement devices may be used in carefully selected patients with mild to moderate disease or continuous positive airway pressure intolerance.

Effective care extends beyond diagnosis and should include multidisciplinary antenatal planning, peripartum risk mitigation, and structured postpartum reassessment. Further prospective studies are needed to clarify which maternal and neonatal outcomes are most responsive to treatment and how screening and therapy can be optimally individualized during pregnancy.
